# A circular tri-trophic system incorporating plants, fish, and insects turns waste into a resource: case study with the cultivation of cucumber

**DOI:** 10.3389/fpls.2025.1638443

**Published:** 2025-10-14

**Authors:** Efi Levizou, Anastasia Mourantian, Michalis Chatzinikolaou, Maria Feka, Ioannis T. Karapanagiotidis, Elena Mente, Christos G. Athanassiou, Konstantinos A. Kormas, Nikolaos Katsoulas

**Affiliations:** ^1^ Department of Agriculture Crop Production and Rural Environment, University of Thessaly, Volos, Greece; ^2^ Department of Ichthyology and Aquatic Environment, University of Thessaly, Volos, Greece; ^3^ Department of Veterinary Medicine, Aristotle University of Thessaloniki, Thessaloniki, Greece

**Keywords:** aquaponics, greenhouse cultivation, photosynthesis, chlorophyll fluorescence, sustainability, soilless cultivation, circularity

## Abstract

**Introduction:**

Circular economy principles are key to enhancing agricultural sustainability and efficiency. In this context, a tri-trophic circular system comprising three types of organisms (plants, insects and fish) that feed each other has been implemented. The nutritional loop involved: i) fertilizing cucumbers with water containing fish metabolic waste through a recirculating water system known as aquaponics; ii) feeding black soldier fly larvae plant pruning residues and fruit; and iii) feeding tilapia fish insect larvae after they have been transformed into insect meal and incorporated into aquafeed. This study aimed to comprehensively evaluate cucumber production in this circular system by investigating key physiological, growth, and yield parameters, and assessing resource use efficiency.

**Methods:**

We implemented in total three treatments, comparing conventional hydroponics (HP) as control, and two aquaponics variants: a) conventional coupled aquaponics (CAP), where water recirculates between crops and fish tanks, so crops receive only fish-derived nutrients; and b) decoupled aquaponics (DCAP), where fish-derived water is enriched with fertilizers to meet the crops' nutritional needs.

**Results:**

DCAP showed similar performance to the HP control, and both outperformed the CAP in terms of plant physiological/functional traits, fruit yield, and biomass accumulation. CAP treatment reduced total aerial biomass accumulation by 57% compared with the HP control, while DCAP increased it by 14%. The enhanced performance exhibited by DCAP can be attributed to its efficient photosynthetic apparatus and optimal leaf nutrient status. Conversely, CAP resulted in a decline in nutrient levels in irrigation water relative to HP and DCAP, which led to significantly decreased leaf concentrations of potassium and phosphorus (2.5 and 1.5 times lower than HP, respectively, by the end of the experiment). This triggered a series of responses, including a down-regulation of the photosynthetic process and a reduced photochemical activity. DCAP exhibited increased fertilizer use efficiency by 76% over HP, achieving a similar fruit yield with reduced fertilizer inputs.

**Discussion:**

In conclusion, DCAP proved to be highly productive, overcoming the limitations observed in CAP, while offering increased environmental and economic advantages compared to HP. The circular tri-trophic system's performance demonstrated its efficacy in harnessing synergies to optimize resource use and ensure high productivity and self-sufficiency.

## 1 Introduction

The incorporation of circular economy concept into agricultural production is a key area of research aiming to increase the sustainability of relevant sectors (Marques et al., 2025). There is a growing interest in finding practical ways to manage all kinds of waste and by-products from fields and greenhouses, with the goal of valorizing them rather than discharging them (Belardi et al., 2025; Martínez-Guillén et al., 2025).

Aquaponics is the combined cultivation of fish and crops in a recirculating water system. It has been proposed as an alternative cultivation method for greenhouses because it is highly sustainable, offering dramatic water savings and minimal chemical inputs (Bernstein, 2011; Krastanova et al., 2022). In theory, the metabolic products of fish would be sufficient to meet the nutritional requirements of crops in a balanced system where the density of fish and crops is well-matched (Yep and Zheng, 2019). However, conventional aquaponics has specific limitations, mainly because, although fish effluent typically provides adequate nitrogen levels, it often contains suboptimal levels of essential elements such as potassium, phosphorus, and iron. K deficiency is the most severe and is mainly attributed to the low K content of the fish feed, which is the only source of nutrients in the conventional aquaponics system (Krastanova et al., 2022). These deficiencies compromise the productivity and quality of crops, especially high-nutrient-demanding greenhouse crops such as tomatoes and cucumbers (Aslanidou et al., 2023, 2024). Nevertheless, the numerous environmental benefits of aquaponics should be exploited, and its increased sustainability should not be overlooked. Thus, much effort has been devoted to optimizing aquaponics to increase productivity while maintaining environmentally friendly characteristics.

One variant that is gaining research interest is the decoupled system (Monsees et al., 2019; Mourantian et al., 2023). Conventional aquaponics involve a single cycle in which water circulates from the fish tanks to the crops and back again (coupled system). In the decoupled system, the water from the fish tanks is enriched with small amounts of fertilizers before reaching the plants, thus addressing their needs and achieving yields similar to those of conventional hydroponics (Aslanidou et al., 2023, 2024).

Increasing the sustainability of farmed fish feed production is also a goal for optimizing the ecological footprint of aquaponics. Currently, fish meal and fish oil are the main sources of protein and are primarily derived from marine forage fish, although an increasing percentage of fishery and aquaculture by-products is contributing to their global production (Hua et al., 2019). As marine forage fish stocks for fishmeal production are reaching their ecological limits, the search for suitable and sustainably produced alternative protein sources for farmed fish feeds is imperative (Quang Tran et al., 2022). Insect meal is gaining attention as a sustainable substitute for fishmeal in farmed fish due to its favorable nutritional profile (Limbu et al., 2022; Karapanagiotidis et al., 2023). Insects are rich in protein (40–70%), which is considered of high quality due to high amounts of amino acids deemed indispensable for fish nutrition (Nogales-Mérida et al., 2019). Insects are also a good source of lipids, vitamins and minerals (Henry et al., 2022). One of the most used species is the black soldier fly (BSF, *Hermetia illucens* L.), which has 60% protein on a dry basis and a wide range of feeding preferences that fit well with the circular economy approach (Karapanagiotidis et al., 2023). Due to these traits, along with a short life cycle and small rearing requirements, BSF has great potential for industrial-scale rearing providing the opportunity to incorporate sustainable, insect-based proteins into fish feed. For all these reasons, the insect-based aquafeed has recently become a loop of aquaponics systems (Stathopoulou et al., 2022; Bordignon et al., 2024; Pinho et al., 2024).

**Figure 1:**
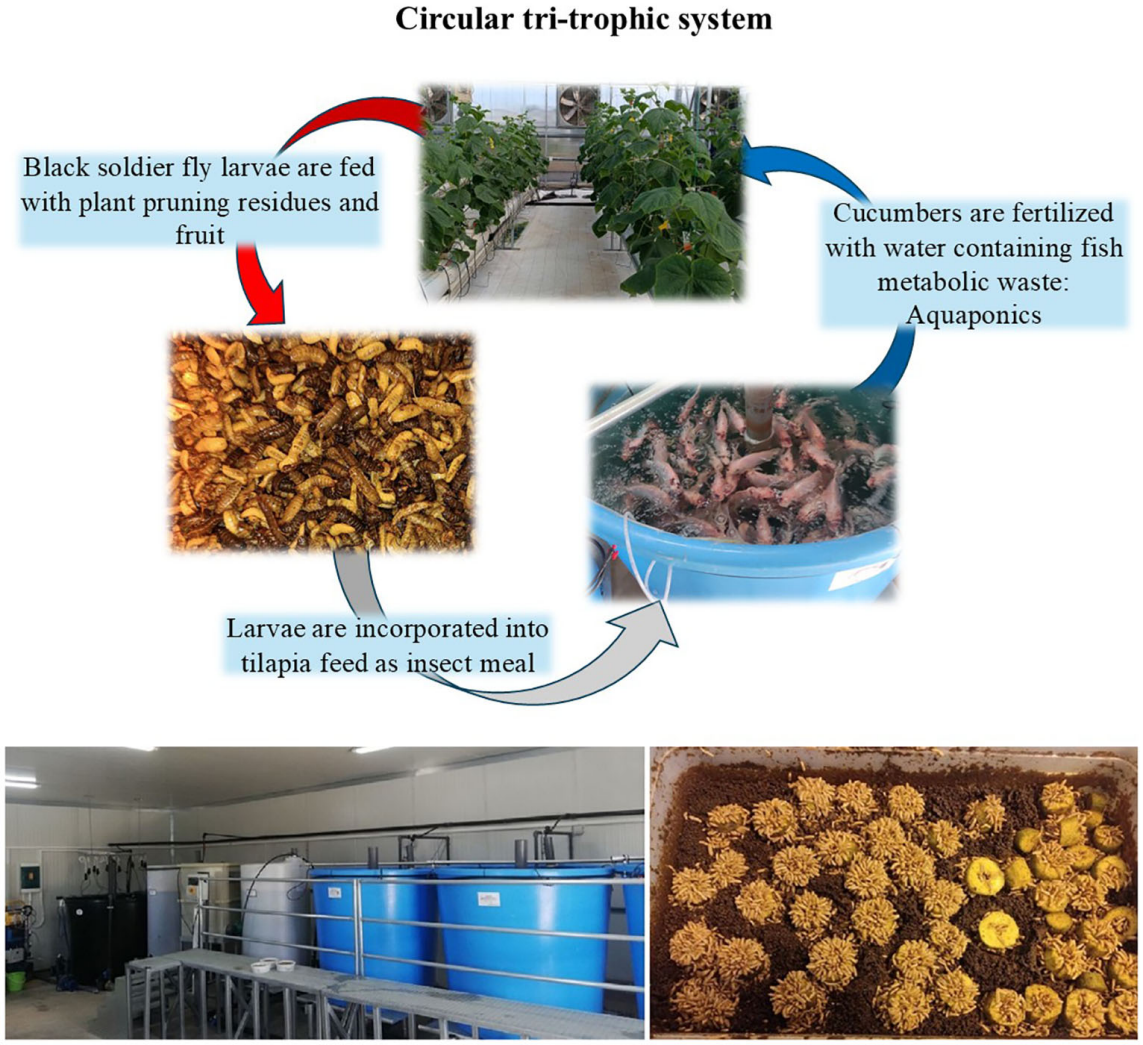
Overview of the circular tri-trophic system (upper illustration). In the lower layer, picture of the RAS system is on the left, with the fish tanks (blue), the mechanical and biological filters (white tanks) and the sump tank (black). In the right picture, BSF larvae feed on cucumber, starting from the soft and juicy inner part of the fruit.

In the present study we aimed at incorporating all the above-described loops into a single production system. We present a circular, tri-trophic system that includes three types of organisms -plants, fish and insects- that feed each other (illustrated in **Figure 1**). Our goal is to apply the principles of the circular economy to the agricultural production by reusing nutrient-rich materials that have so far been considered waste. By joining the cycle of nutrition, the residues or by-products of the metabolism of one organism will become food for the next, turning them from waste into a resource. Insects, notably BSF, feed the fish through the production of insect meal being incorporated into the feed. Fish are linked to plants through aquaponics, excreting their metabolic products to support plant nutrition. Plant residues resulting from the high-wire farming system (e.g., pruning residues, cut fruits) provide food for BSF.

The evaluation of this circular tri-trophic system in terms of its effects on greenhouse cucumber production was the primary objective of this study. We have chosen cucumber as experimental plant because it is a common greenhouse crop with high-nutrient demands. We used hydroponics as the control group and also tested two aquaponics systems, coupled and decoupled. Our comprehensive evaluation included crop growth and yield characteristics as well as functional traits. The latter are often overlooked in the relevant literature, although the functional performance, e.g. the photosynthetic process, regulates growth. Moreover, elucidating the mechanisms underlying yield responses provides valuable insights for optimizing productivity. Finally, the estimation of resource use efficiency was conducted, in terms of fertilizer and water use efficiency (FUE and WUE, respectively).

## 2 Materials and methods

### 2.1 Aquaponic unit

The experiment was carried out in the pilot-scale aquaponics greenhouse of the University of Thessaly, located in Velestino (39°44′ N, 22°79′ E), Central Greece. The greenhouse facility consisted of the hydroponic plant growing area (360 m^2^) and the environmentally controlled chamber where the recirculating aquaculture system (RAS) operated (80 m^2^). A comprehensive description of the hydroponic and RAS subsystems, as well as environmental management within the RAS compartment and the entire greenhouse, can be found in previous articles (Aslanidou et al., 2023; 2024). Briefly, irrigation, fertigation and greenhouse climate management were performed and controlled automatically by software (Argos Electronics, Evia, Greece). Climate control was based on temperature, humidity and radiation sensors readings (iMETOS^®^sm meteorological station, Pessl Instruments, IMT180, Weiz, Austria). Ventilation was provided by roof openings when the temperature and relative humidity exceeded 21°C and 85%, respectively. Heating was provided by air heaters at 18°C and the fan and wet pad evaporative cooling system was activated at a temperature set point of 26°C.

The plant cultivation area consisted of 18 channels of 8.5 m length each, installed at a height of 50cm above the ground. In each channel, 8 perlite slabs were placed (particles diameter 1–5 mm, Perterra, NORDIA S.A., Athens, Greece) into which the cucumber plants were transplanted and watered by drippers (5 per slab). The irrigation rate fluctuated daily according to plants’ needs, and on a larger timescale according to their developmental stage, as detailed in Aslanidou et al. (2024).

The RAS included the following: (i) 3 fish tanks of 1300 l each, (ii) a mechanical filter tank (Rotary Drum Filter, ProfIDrum B.V., Scandia, MN, USA), (iv) a biofilter tank and (v) a sump tank of 2,500 l, as depicted in **Figure 1**. The mechanical filter belongs to the drum filter category and is used for the removal of insoluble fish solid feces. Inside the biofilter there are ceramic rings (15 mm) and K1 (inside Kaldness 1 mm) which serve as a substrate for nitrifying bacteria growth (Prodibio, Biodigest, Marseille, France). A system of sensors for temperature, pH, electrical conductivity (EC), and dissolved oxygen (DO) was installed in the sump tank, where fish water was collected, to enable continuous monitoring of water quality characteristics (pH/EC/O_2_-measuring transducer, GHM-Greisinger, Regenstauf, Germany).

### 2.2 Fish rearing

Red tilapia (*Oreochromis* spp.) fish were reared in the RAS. At the beginning of the experiment, 329 fish were weighed and distributed in the three tanks according to the equation of aquarium carrying capacity proposed by Hirayama (1974). The total initial biomass was 141.1 kg; 160 fish (43.7 kg), each weighing up to 450g each were placed in the first tank, 102 fish (48.6 kg) up to 700g each in the second tank, and 67 fish (48.8 kg) up to 1 kg each in the third tank. The fish in each tank were fed at a rate corresponding to 1% of their total body weight daily. Fish feed included insect meal derived from the insects farmed on-site (details in the relevant paragraph); the experimental diet formulation, and proximate composition are presented in **Table 1**. Analyses of proximate composition, as well as the preparation of the pelleted feed, were performed as described by Karapanagiotidis et al. (2023).

**Table 1:** Formulation as % of incorporation of each dietary ingredient, and proximate composition, expressed as % of feed dry weight (DW) of the fish feed.

Ingredient composition (% incorporation)			
Fishmeal	17.00	Vitamins and minerals, premix*	0.70
BSF larvae meal	10.00	Monocalcium phosphate	0.30
Wheat meal	24.00	Vitamin C	0.25
Rapeseed meal	19.00	Vitamin E	0.25
Corn gluten meal	13.00	L-Lysine	0.50
Sunflower meal	9.00	DL-Methionine	0.50
Soya oil	5.50		
Proximate composition (% DW)			
Moisture		9.68±0.17	
Crude protein		38.15±0.28	
Crude lipid		10.23±0.01	
Ash		6.59±0.05	
Gross energy (MJ/kg)		19.15±0.31	

*Vitamin and mineral premix, custom-made (per kg of mixture): Vitamins: E, 58.3 g; K3, 3.3 g; A, 1500 IU/g; D3, 200 IU/g; B1, 3.3 g; B2, 6.6 g; B6, 3.3 mg; B12, 10 mg; folic acid, 3.3 g; biotin, 100 mg; inositol, 40 g; C, 33.3 g; nicotinic acid, 16.6 g; pantothenic acid, 13.3 g. Minerals: Co, 170 mg; I, 248 mg (Ca(IO3)2); Mn, 10 g (MnO); Zn, 33 g (ZnO); Ca, 235 g; Se, 2.5 mg (Na_2_SeO_3_); Na, 247.5 mg (Na_2_SeO_3_); Fe, 2 g; Mg, 121.3; Cu, 0.8 g.

The physicochemical parameters of the water in all fish tanks were measured daily; pH, EC and DO were measured with a portable sensor (HQ40d, Hach, Loveland, CO, USA). The DO level was maintained at 7.0 mg l^-1^ (± 0.3 mg l^-1^), with air being supplied by an air blower at a rate of 100 m^3^ h^-1^, and 22 air diffusers. Temperature was measured with a portable sensor (Combo pH-EC-TDSTemp, 98130 Hanna Instruments, Woonsocket, RI, USA) and maintained at 23 (± 0.4)°C.

At the end of the experiment, the total biomass of fish was measured again and Specific Growth Rate (SGR) and Feed Conversion Efficiency (FCR) indices were calculated as follows:


$$SGR\;(\%\;/day)=\frac{\ln(final\;weight)-\ln(initial\;weight)}{\Delta t}\times 100$$

$$FCR=\frac{Consumed\;feed\;(g)}{Final-initial\;weight\;(g)}$$

The EU Directive 2010/63/EU concerning the protection and welfare of experimental animals was followed in all the steps of the experiment. FELASA-accredited scientists implemented all the animal-related experimental procedures (functions A-D). The experimental protocol was approved by the Animal care and Use Ethics Committee (approval number 242627/28-05-2024) and conducted at the registered experimental facility (EL-43BIO/exp-02) at the University of Thessaly.

### 2.3 Insect breeding unit

The insect breeding subsystem was installed in a 32 m^2^ climate-controlled chamber, located next to the pilot aquaponics greenhouse. Throughout the rearing period, the temperature was kept close to 23°C and the relative humidity close to 40%, using climate controlling equipment. The initial BSF adult insects were derived from a population reared at the premises of the Laboratory of Entomology and Agricultural Zoology of the University of Thessaly. Adult insects were mated inside a love cage (70x70x86 cm), illuminated by an LED mating lamp. The adult flies laid their eggs in the gaps of custom-made wooden structures placed in shady spots inside the love cage. The eggs were then collected and placed in small boxes filled with chicken feed which met the initial nutritional needs of the newly hatched larvae. After one week, the larvae were transferred to a larger box and fed with plant residues (pruned leaves and stems, and cucumbers) from the cucumber cultivation. The moisture of the feed was adjusted with water according to the larvae’s preferences (**Figure 1**). Once the larvae reached an optimal size of approximately 2–2.5 cm, they were collected, oven-dried at 40°C for 5 hours and then vacuum-dried for 24 hours. Thereafter, the samples underwent a milling and sieving process, resulting in a particle size of less than 1 mm. This insect meal was then incorporated into the fish feed. A small portion of the initial larvae population was left alive to complete its biological cycle. The nymphs’ pupation process took place in a dark cage (66x75x67 cm) to renew the adult fly population.

### 2.4 Experimental design

**Figure 2:**
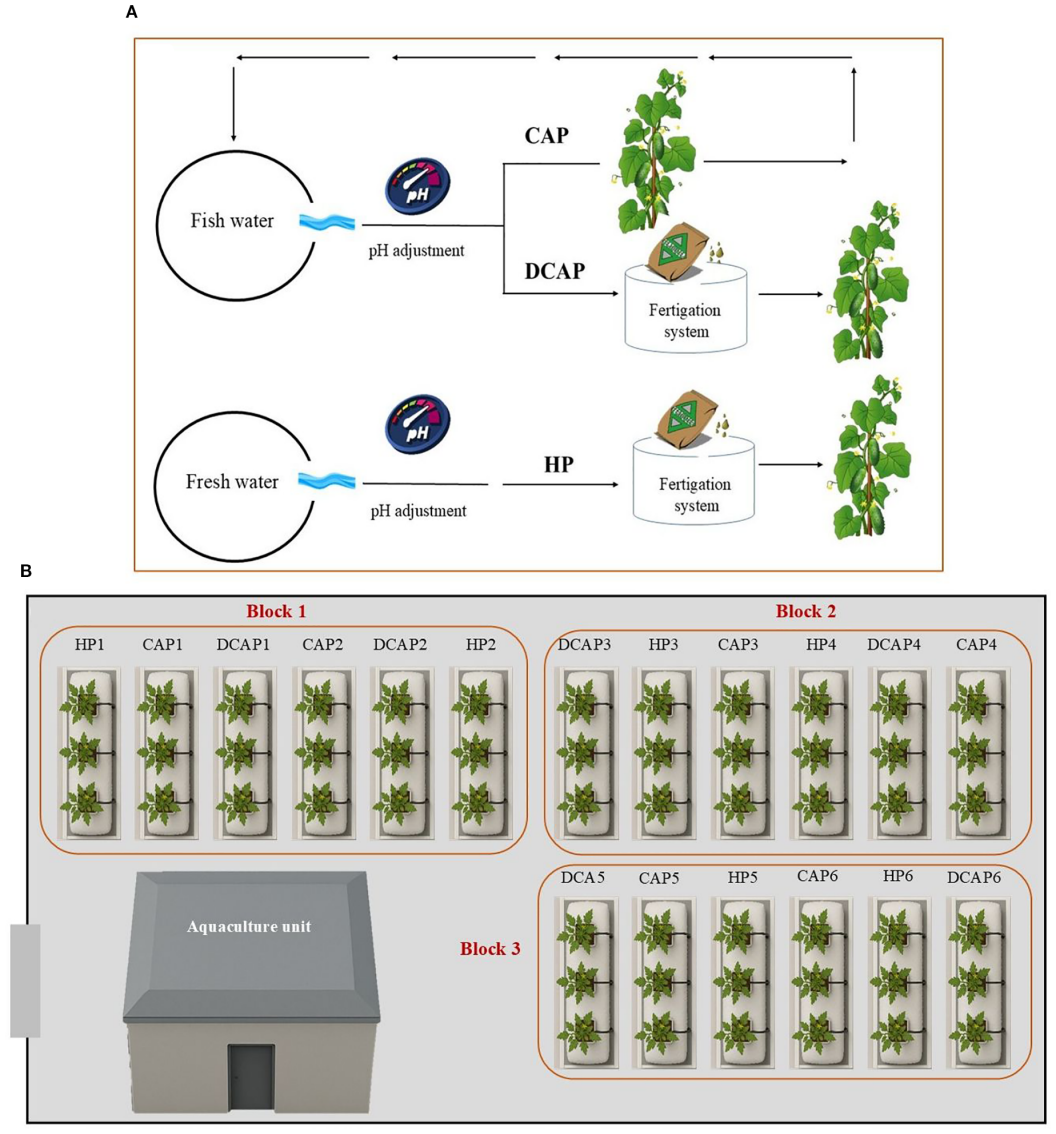
Schematic depiction of the experimental layout; **(A)** an illustration of the three treatments and **(B)** treatment distribution in the channels of the plant cultivation area. In the CAP treatment the fish-derived water after the pH adjustment was driven to the hydroponic unit and returned to the fish tanks. In the DCAP treatment, the fish-derived water underwent pH adjustment and fertilizer amendment before reaching the crops. HP is the conventional hydroponics using fresh water.

A total of 288 cucumber (*Cucumis sativus* var. Columbia) seedlings at the four-true-leaf stage were transplanted at a density of 1.175 plants/m^2^. The cultivation period lasted 90 days. The experimental setup included the following three treatments regarding the irrigation solution (**Figure 2**):

- hydroponics (HP): conventional hydroponic nutrient solution for cucumber cultivation under Mediterranean climatic conditions (Control)
- coupled aquaponics (CAP): fish water with no fertilizer amendments and only pH adjustment
- decoupled aquaponics (DCAP): fish water amended with chemical fertilizers to reach the concentration targets of HP

The HP solution formula was based on Savvas et al. (2013), in which the fertilizers and their concentrations differ according to the phenological stage of the crop, whether vegetative or reproductive (**Table 2**). In order to prepare the DCAP irrigation solution, the concentration of certain nutrients in the fish water was analyzed on a weekly basis. Subsequently, it was enriched with high-purity fertilizers at the proper concentrations to reach the HP target values for each phenological stage. Details regarding the fish water analyses and the nutrient solution calculations are reported in Aslanidou et al., 2023). In the CAP treatment, crops were irrigated directly with fish water after adjusting the pH to the 6.0–6.5 range to increase nutrient availability. Then, the water from the CAP channels was driven back to the fish tanks to close the circle.

**Table 2:** Irrigation solution formula for HP for two different phenological stage of the crop, vegetative and reproductive, according to Savvas et al. (2013).

Macronutrients (mmol/L)			Micronutrients (μmol/L)		
	*Vegetative stage*	*Reproductive stage*		*Vegetative stage*	*Reproductive stage*
NO_3_^-^	14.75	13.75	Fe	15	15
NH_4_^+^	1.40	1.40	B	25	25
P	1.25	1.15	Cu	0.80	0.80
K	6.20	7.20	Mn	10	10
Ca	4.15	3.40	Zn	5	5
Mg	1.60	1.40	Mo	0.50	0.50
S	1.30	1.40			

Regarding replication, the greenhouse was divided into three identical blocks, each with 6 hydroponic channels, which were randomly allocated to the three treatments. Thus, each treatment was replicated 6 times (2 channels per block*3 blocks) and there were 96 plants/treatment.

### 2.5 Crop measurements

#### 2.5.1 Growth parameters and fruit yield

A total of three plant harvests were performed during the experiment (Day (D)25, D50, D90). At each harvest, 6 plants per treatment (except roots and fruits) were removed in order to measure the fresh and dry weights of leaves and shoots separately. After the immediate separation of the aerial plant parts, their fresh weight was recorded and then they were oven-dried at 70°C until they reached constant dry weight.

Fruit yield was determined on a weekly basis. After D20 and every 2–3 days, fruits at the commercial size were harvested, separately for each treatment, and immediately weighed to record their fresh weight. The total weight per treatment per block was considered the weekly yield (avg±se), and it was expressed per area unit to facilitate among-treatments comparisons. The cumulative yield presented in the Results section was calculated by adding the weekly recordings of total cucumber production per treatment, given as kg.

#### 2.5.2 Chlorophyll a fluorescence

Chlorophyll *a* fluorescence measurement was performed at three time points during the experiment (D20, D45, D80) on pre-darkened leaves using the fluorometer FluorPen FP 110 (PSI, Photon Systems Instruments, Czech Republic). All measurements were performed on clear days between 10:00 and 11:30 a.m. One mature leaf from 15 plants/treatment, positioned in the middle of the plant was selected and dark-adapted for 30 minutes, with a leaf clip. Afterwards, the leaf was illuminated for 2 sec with 3,000 µmol photons m^−2^ s^−1^ at 650 nm. FluorPen 1.1 software was subsequently used for the extraction of the OJIP parameters. A brief interpretation of the parameters derived from fluorescence transients according to Strasser et al. (2004) are summarized in **Table 3**. The results presented in the relevant section were normalized to the values of HP, serving as control.

**Table 3:** Chlorophyll fluorescence parameters derived and calculated from the OJIP data (from Strasser et al., 2004).

Fluorescence parameters	
F_M_	Maximal fluorescence from a dark-adapted leaf
F_V_	Maximal variable fluorescence from a dark-adapted leaf. FV=FM−F0
F_V_/F_M_	Maximum quantum efficiency of PSII photochemistry
Vi	Relative variable fluorescence at phase I of the fluorescence induction curve
1-Vi	Measure of relative amplitude of the IP phase in OJIP transient, related to the size of the pools of final PSI electron acceptors
1/Vi	Relative measure of the pool size of final electron acceptors of PSI
ABS/RC	Absorption flux (for PSII antenna chls) per reaction center (RC)
TR_0_/R	Trapped energy flux per RC (at t =0)
DI_0_/RC	Dissipated energy flux per RC (at t =0)
PI_TOTAL_	Performance index total for energy conservation from photons absorbed by PSII to the reduction of PSI end acceptors
PI_ABS_	Performance index for energy conservation from photons absorbed by PSII antenna
Sm	Normalized area above the OJIP curve

#### 2.5.3 Leaf gas exchange

Gas exchange measurements were performed on sunny days (D20, D45, and D70) between 9:30 and 11:30 a.m. using a portable system (LI-6400/XT, LI-COR, Lincoln, NE, USA). On each measurement day, one mature leaf was selected from the middle part of the plant, from10 plants/treatment. The conditions inside the leaf chamber were set to resemble the prevailing conditions in the greenhouse and were kept constant to avoid any fluctuations during the measurements and among the treatments. Specifically, the settings were 400 ppm CO_2_ (with the 6400–01 CO_2_ Injector), 23°C, and photosynthetic photon flux density of 600 μmol m^-2^ s^-1^ (LED lamb 6400-02B). The net photosynthetic rate (A_N_, µmol m^−2^ s^−1^), transpiration rate (Tr, mmol m^−2^ s^−1^), stomatal conductance (g_s_, mol m^−2^ s^−1^) were recorded. The intrinsic water use efficiency (iWUE, µmol mol^−1^) was estimated as the ratio of A_N_ to g_s_.

#### 2.5.4 Leaf nutrient concentrations

Dry leaf tissue of 6 plants/treatment derived from the three plant harvests (D25, D50 and D90) was used to conduct the leaf elemental analyses for the macronutrients N, P, K (expressed as mg g DW^-1^). The details of each procedure are extensively described in Aslanidou et al. (2023). Briefly, the concentration of N was determined with the Kjeldahl method (behr Labor-Technik, Germany). For [P] and [K] determination an acid extraction was performed to leaf tissue with 20 ml of HCl (6%) followed by dilution with water up to 50 ml. [P] was then measured, after the blue color development using ammonium vanadomolybdate/ascorbic acid, with a photometer (dual-beam, UV-Visible, UV1900, Shimadzu, Japan). Finally, [K] was determined with a flame photometer (Jenway PFP7, Cole-Palmer, UK). The calibration curves for [P] and [K] determination are presented in the supplementary material (**Supplementary Figure S1**).

### 2.6 Resources use efficiency

The WUE of the three treatments was calculated as the ratio of the total fruit yield (in kg) to the total volume of the irrigation solution consumed (in m^3^) throughout the entire cultivation period. Likewise, the FUE was calculated as the ratio of the total fruit yield (kg) to the total quantity of fertilizers consumed (in kg) during the cultivation. FUE was estimated for HP and DCAP, since the CAP treatment received no fertilizers.

### 2.7 Statistical analysis

The data were analyzed using the free statistical software JASP 0.18.3 (JASP Team 2021 Computer Software). Statistical analyses of among-treatments differences of each parameter at each measurement date were performed with one-way ANOVA followed by Tukey *post-hoc* tests. The nonparametric Kruskal-Wallis test was used in cases where ANOVA pre-requisites were not met. The sample size for each measured parameter was chosen according to our previous published experiments in a similar setup within the pilot aquaponics greenhouse, and relevant articles. Based on the sensitivity of each method, the chosen sample sizes balance feasibility with analytical rigor, minimizing the risk of Type II errors.

## 3 Results

### 3.1 Physicochemical characteristics of the irrigation solutions and fish growth

**Table 4** summarizes key physicochemical parameters of the three irrigation solutions tested in the cucumber cultivation. The pH was strictly regulated within the range of 6.0 to 6.5 to ensure maximum nutrient availability for the crops. The EC showed statistically significant increases in HP and DCAP compared to CAP, due to the amendment of fertilizers. However, the values of 1.92 and 2.05 dS m^-2^ measured in HP and DCAP respectively are within the typical range for cucumber soilless cultivation.

**Table 4:** Physicochemical parameters of the irrigation solution of the three treatments (Avg±SE).

Treatments	pH	EC	NO_3_^-^	NH_4_^+^	PO_4_^3-^	K^+^	Na^+^	Ca^2+^
HP	6,32±0,10	1,92±0,08^a^	9,08±0,59^a^	0,35±0,08^a^	0,92±0,12^a^	4,49±0,35^a^	1,27±0,10	3,76±0,25^a^
CAP	6,48±0,12	1,11±0,02^b^	4,64±0,19^b^	0,03±0,01^b^	0,28±0,01^b^	1,13±0,06^b^	1,18±0,10	1,76±0,09^b^
DCAP	6,31±0,10	2,05±0,05^a^	8,85±0,49^a^	0,70±0,10^a^	1,01±0,15^a^	5,19±0,20^a^	1,35±0,10	3,59±0,20^a^

The different letters denote statistically significant differences between treatments for each parameter (p ≤ 0.05). The units are dS m^-2^ for EC and mmol l^-1^ for the nutrient concentrations.

In all nutrient concentrations determined throughout the experiment, CAP exhibited the lowest values, with statistically significant differences appearing in all but Na^+^. The decline in CAP irrigation solution in comparison with the control HP was twofold in NO_3_^-^ and Ca^2+^, threefold in PO_4_^3-^, 4-fold in K^+^, and 10-fold in NH_4_^+^. In all cases, the HP and DCAP treatments showed similar levels, thus no statistically significant differences were recorded between them.

Fish biomass was increased from 141.1 kg at the beginning of the experiment to 159.8 kg at the end, showing a weight gain of 18.7 kg along the three-month period. The SGR was estimated at 2.1% day^-1^ and the FCR at 1.56.

### 3.2 Crop growth

**Figure 3:**
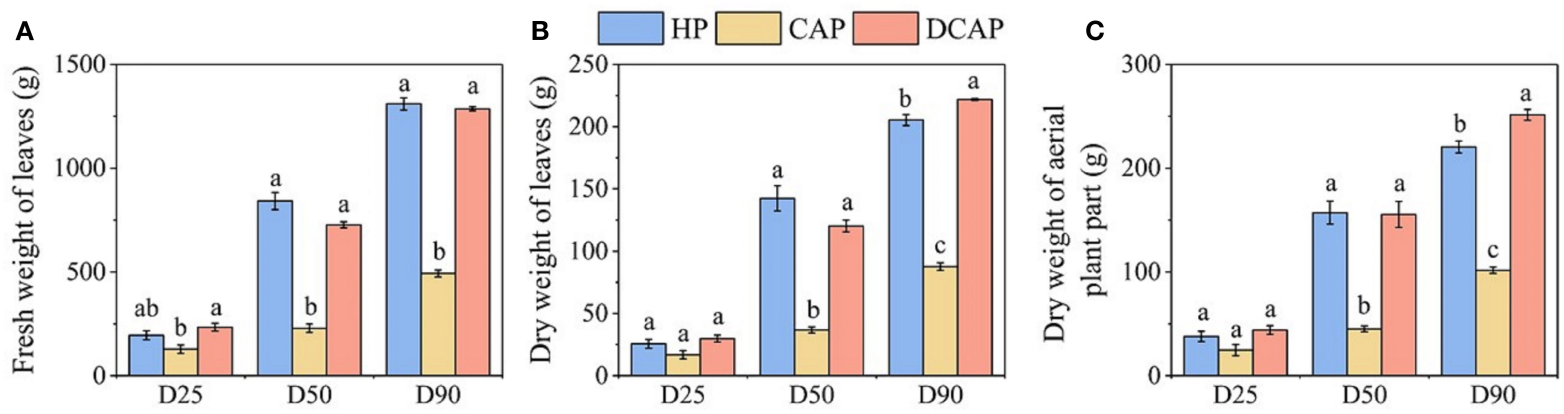
Fresh **(A)** and Dry **(B)** weight of cucumber leaves, and Dry weight of the aerial plant part **(C)** (Mean±SE) measured at different time points during the experiment. Different letters denote statistically significant differences among treatments at each measurement date (p ≤ 0.05).

Biomass accumulation in the aerial part of cucumber was determined at three timepoints, i.e. on D25, D50 and D90 and is shown in **Figure 3**. The fresh and dry weights of leaves (**Figures 3A, B**), as well as the total dry weight of the aerial parts, including stems (**Figure 3C**), followed the same profile. They were considerably declined under the CAP treatment compared with both HP and DCAP. Statistically significant differences were recorded already in the first month of the experiment (D25) in leaves’ fresh weight, as well as in the subsequent measurements of the dry biomass of leaves and the entire aerial part. Indicatively for the dry biomass of leaves, the inferiority of CAP was mirrored in the 35% decrease compared to HP on D25, the 74% and 57% on D50 and D90 respectively. The weights of leaves and total aerial part in HP and DCAP treatments were comparable. The only statistical difference appeared at the end of the experiment, when the DCAP outperformed the control by 10% for the leaves and by 14% for the total dry weight.

### 3.3 Crop yield

**Figure 4:**
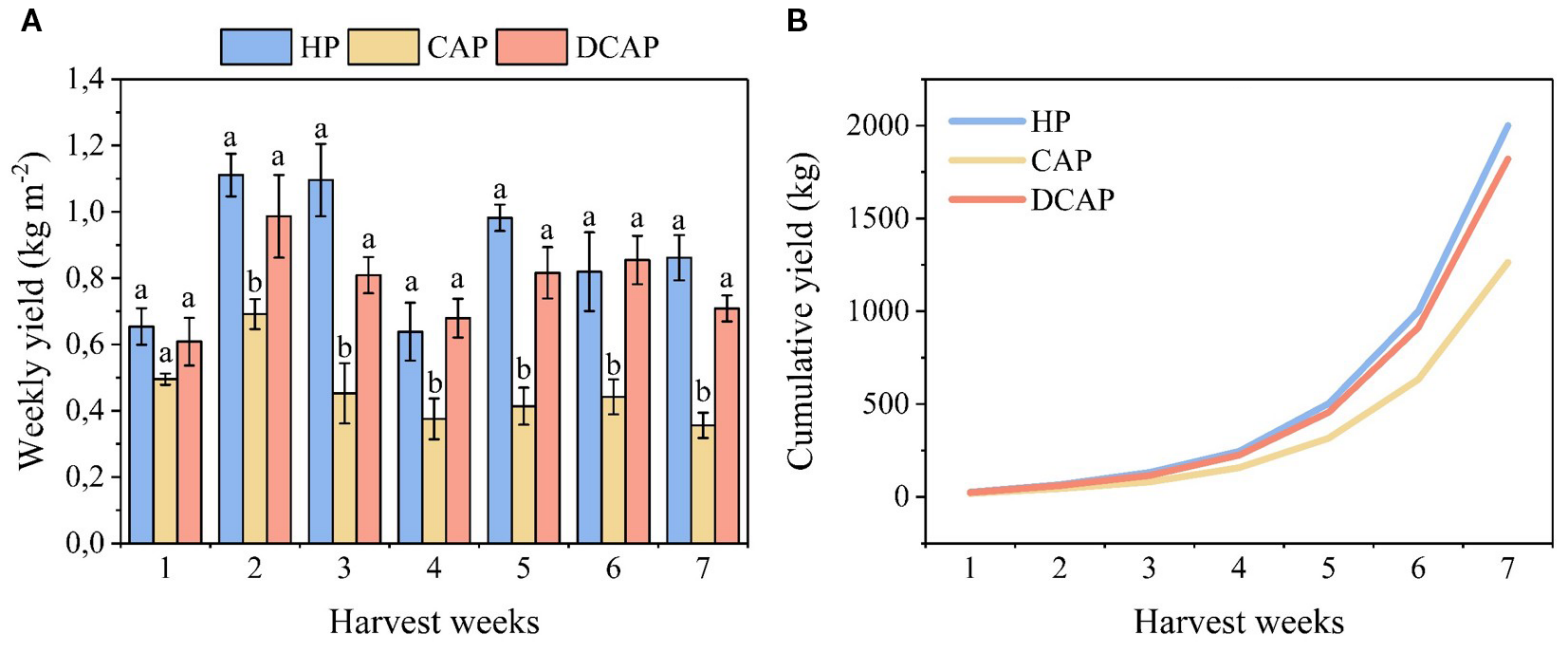
Yield parameters of cucumber; weekly production expressed in kg m^-2^ **(A)** and cumulative yield along the entire cultivation period in kg **(B)**. The different letters in (a) denote statistically significant differences among treatments in each weekly assessment (p ≤ 0.05).

The effects of the three treatments on cucumber yield is depicted in **Figure 4** as the weekly production expressed in kg m^-2^ (**Figure 4A**), and as the cumulative production in kg of product along the whole cultivation period (**Figure 4B**). Although a fluctuation of cucumber production was obvious on a weekly basis, the general trend of among-treatments differences remained consistent throughout the experiment (**Figure 4A**). There was no statistically significant difference between HP and DCAP as their values were quite close in all weekly harvests. Yet, non-significant differences ranging from 17% to 26% appeared in favor of HP in the 3^rd^, 5^th^ and 7^th^ weeks. CAP yield was consistently lower, showing a stable decrease of 57%-58% compared to HP.

The cumulative yield for the entire cultivation period reflects the differences among the treatments: HP produced 6.16 kg m^-2^ cucumbers, DCAP reached 5.46 kg m^-2^ (11% less than HP), and CAP produced 3.23 kg m^-2^ (47.6% less than HP). All values refer to the total yield (kg) expressed per m^-2^, over the entire period.

### 3.4 Chlorophyll a fluorescence

**Figure 5:**
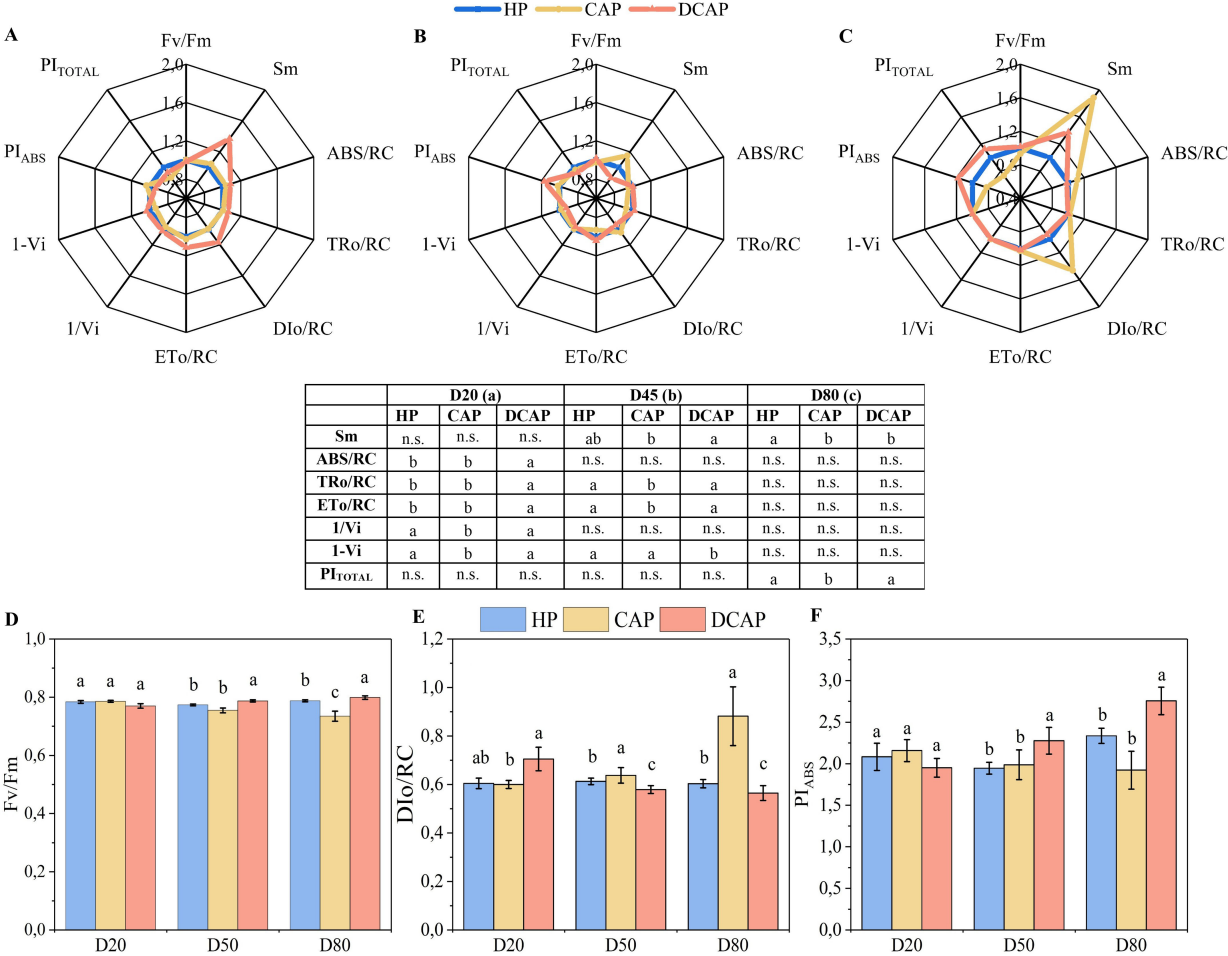
Radar plots depicting the changes in the values of chlorophyll fluorescence parameters from the JIP-test, for D20 **(A)**, D50 **(B)** and D80 **(C)**. The values are normalized to HP values (regarded as 1.0). The results of statistical analyses are presented in the bottom table, where n.s. means non-significant differences among treatments, and the different letters indicate statistically significant differences among treatments in each measurement date (p≤0.05). In the bottom panel, the Fv/Fm **(D)**, DIo/RC **(E)** and PIABS **(F)** (Mean ± SE) are presented in bars for the whole experimental period, where the different letters indicate statistically significant differences among treatments in each measurement date (p≤0.05).

The parameters of *in vivo* chlorophyll a fluorescence are presented in radar plots (**Figure 5**), accompanied by the table reporting the statistical analysis. As previously mentioned, the plotted values of the DCAP and CAP parameters are normalized to the HP values. On D20 (**Figure 5A**), statistically significant differences appeared between HP and DCAP treatments only in parameters related to energy fluxes per PSII reaction center (ABS/RC, TRo/RC, ETo/RC). In contrast, CAP differed from HP in the PSI-related parameters (1-Vi and 1/Vi) and from DCAP in all the aforementioned parameters (**Figure 5A**).

On D45 (**Figure 5B**), DCAP showed a superior efficiency of its photosynthetic apparatus, which was depicted in both higher maximum quantum yield of PSII photochemistry (Fv/Fm) and the index of total photosynthetic efficiency (PI_TOTAL_). At this time point, CAP presented an increase in Sm, denoting the relative pool size of primary electron carriers (Q_A_) of PSII, which is considerably enlarged at the final measurement of D80 (**Figure 5C**).

Notably, on D80, CAP exhibited increased DIo/RC, i.e. the dissipated energy flux per reaction center, compared to DCAP, a trend evident since D45 (**Figure 5E**). All the performance indices (Fv/Fm, PI_TOTAL_ and PI_ABS_) were significantly decreased on D80 in CAP, in contrast to DCAP, with the latter exhibiting the highest values (**Figures 5D, F**).

### 3.5 Gas exchange

**Figure 6:**
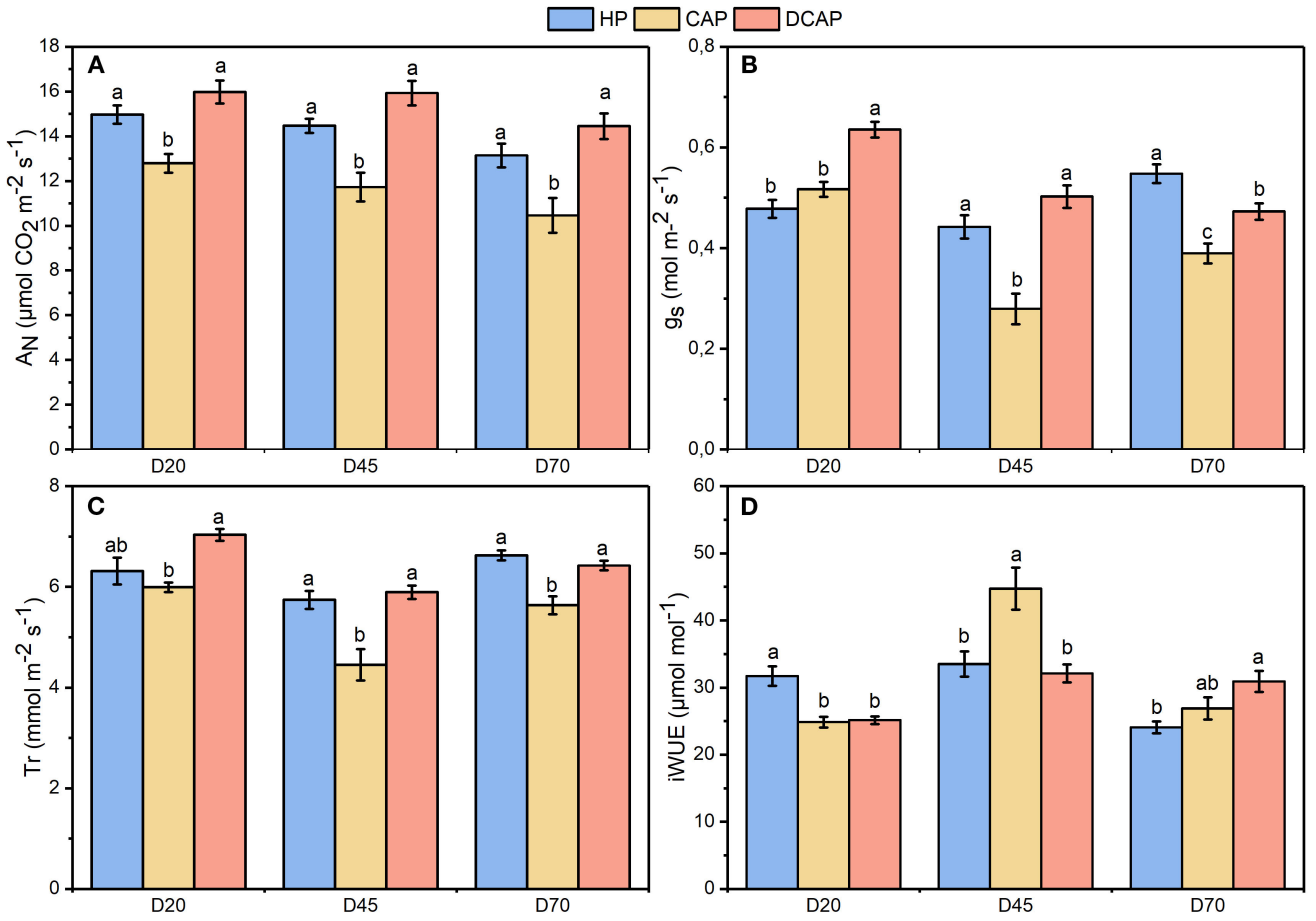
The dynamics of gas exchange parameters during the experimental period: **(A)** A_N_, net photosynthetic rate; **(B)** Tr, transpiration rate; **(C)** g_s_, stomatal conductance; **(D)** iWUE, intrinsic water use efficiency (Mean±SE). Different letters indicate statistically significant differences among treatments at each measurement date (p ≤ 0.05).

The effects of the different treatments on gas exchange were evident from the first measurement, as depicted in **Figure 6**. Net photosynthetic rate (**Figure 6A**) remained high and consistent for both HP and DCAP, ranging from 13 to 16 μmol CO_2_ m^-2^ sec^-1^, throughout the experimental period. CAP showed significantly lower A_N_ on all measurement dates compared to both HP and DCAP.

Analogous profile was exhibited by Tr (**Figure 6C**), except for the non-significant difference between HP and CAP on D20. This was ascribed to significantly lower g_s_ recorded on that day on HP plants in comparison to DCAP (**Figure 6B**). On D70 the g_s_ of HP was significantly higher than that of CAP and DCAP.

The aforementioned fluctuations of A_N_ and g_s_ shaped the profile of iWUE, which showed no consistent trend across measurements (**Figure 6D**). HP exhibited significantly higher values on D20 compared to the other two treatments due to a high A_N_ succeeded by lower g_s_. The same picture was presented by CAP in the intermediate measurement (D45), while DCAP’s iWUE remained relatively stable during the experimental period.

### 3.6 Nutritional state of leaves

**Figure 7:**
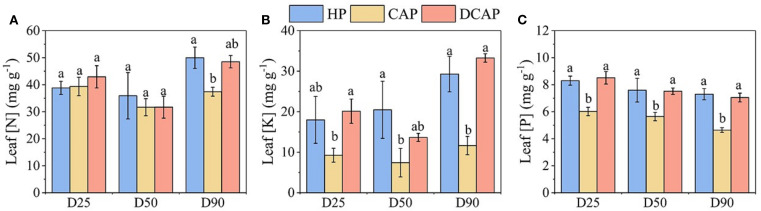
The results of leaf elemental analysis throughout the experiment; leaf N concentration **(A)**, K concentration **(B)** and P concentration **(C)**. Different letters indicate statistically significant differences among treatments for each element and measurement date (p ≤ 0.05).

The nutritional state of cucumber leaves expressed by the concentrations of nitrogen (N), potassium (K), and phosphorus (P) is shown in **Figure 7**. In general, the nutrient content in leaf tissues of HP and DCAP leaves was similar, yet superior to that of CAP plants in most cases. Leaf N content is high and at similar levels for all treatments on D25 and D50 (**Figure 7A**). However, on D80 the nitrogen content of CAP leaves is 35% lower than those of HP and DCAP, a difference that is statistically significant.

The among-treatments differences in K concentrations were pronounced (**Figure 7B**). The [K] in DCAP leaves was two to three times higher than in CAP, with HP showing analogous increases. Notably, the level of [K] in CAP remained almost stable (7.4-11.6 mg g^-1^), in contrast to the other two treatments; an increase of 43% was recorded in HP, and DCAP increased 2.5-fold (29.3 and 33.3 mg g^-1^ in HP and DCAP, respectively) on D80 compared to D50 levels.

Leaf [P] variation was minimal during the experimental period (**Figure 7C**). This resulted in consistent differences among treatments across all measurements. DCAP and HP showed similar levels, while [P] in CAP was significantly lower by 40-60% (D25 and D80, respectively).

### 3.7 FUE and WUE

**Table 5** shows the water and fertilizer use efficiencies obtained in all three treatments. The HP showed the greatest WUE, followed by DCAP, with their difference being only 7%. CAP resulted in decreased WUE by 43.2% compared to the HP control. The FUE was estimated only for HP and DCAP, since no fertilizers were added to CAP. DCAP outperformed the HP, exhibiting a FUE of 59.9, increased by 74.2% in comparison with HP.

**Table 5:** WUE and FUE of cucumber subjected to the three treatments.

Treatments	WUE (kg cucumbers m^-3^ water used)	FUE (kg cucumbers kg^-1^ fertilizers used)
HP	100.5	34.4
CAP	57.1	
DCAP	92.9	59.9

All the components of the indices are expressed per m^2^. In CAP no fertilizers were used, so there is not FUE value.

## 4 Discussion

The tri-trophic system presented in this study integrates into a big circle the already circular aquaponics system (fish-plants), the insect-based feed for fish rearing (insect-fish) and the plant-based feeding of insects (plant-insect) (**Figure 1**). These partial circles are incorporated in our proposed system, in which one organism’s waste and residues become a resource for feeding another organism. This approach fosters system-level circularity, which, in turn, enhances both the resource use efficiency and the sustainability of the crop and fish production system.

Aquaponics is already considered a highly sustainable cropping system. However, bottlenecks regarding crop productivity have emerged during the intensive research of the previous decade (Stathopoulou et al., 2021; Tsoumalakou et al., 2022, 2023b; Mourantian et al., 2023). The inferiority of the coupled aquaponics system is attributed to the reduced nutrient availability in the fish water that was subsequently transferred to the crops. This is consistent with the results of the present experiment, in which the concentrations of all measured nutrients in the irrigation water were found to be significantly lower in the CAP treatment compared to the conventional hydroponics treatment (HP), considered as control (**Table 4**). However, the introduction of decoupled aquaponics ameliorated these deficiencies, resulting in nutrient concentrations that approximate those observed in HP. The relevant literature supports these results; well-designed experiments in respect to fish-crop population matching and appropriate fertilizer inputs succeeded similar levels of nutrients with HP in the irrigation solution (Delaide et al., 2019; Aslanidou et al., 2023).

Fish rearing with BSF-based feed resulted in SGR of 2.1% day^-1^ and FCR of 1.56 (**Table 5**), which are at the upper limits of the typical range for tilapia. Shaw et al. (2022) testing the complete substitution of fishmeal with BSF meal in tilapia feed found a lower SGR (1.76% day^-1^) compared with ours, while their positive control with fishmeal achieved the same as ours (2.1%). A similar profile of differences also holds for the FCR, where both their BSF treatment and positive control achieved lower levels (1.03 and 0.79, respectively) than ours. Likewise, in a recent work of Limbu et al. (2022) a variety of tilapia diets was tested; the complete replacement of fishmeal with BFS larvae meal succeeded the best SGR (2.1 day^-1^) and FCR (1.01) surpassing all other percentages and the positive control, which were at same and lower levels than ours, respectively. Since comparing BSF-based fish feed to typical fishmeal was beyond the scope of this study, the aforementioned studies focusing on this comparison confirm the efficiency of this sub-cycle of our proposed tri-trophic system. Another important indirect conclusion from these studies is that feeding BSF with plant residues and non-commercial cucumbers in the present experiment is effective in producing high-quality insect meal that supports the high growth performance of tilapia in our aquaponics system.

Plant growth and productivity were seriously compromised by the low nutrient availability in CAP irrigation solution, which in turn resulted in lower leaf nutrient contents compared to HP and DCAP (**Figures 3**, **7**). Conversely, DCAP achieved high biomass accumulation and yield, similar to HP, due to efficient nutrient uptake from an irrigation solution with high nutrient availability. Therefore, the growth and productivity responses in both aquaponics treatments are directly connected to nutrient flow and absorption processes. The superiority of DCAP performance over CAP may also be explained through another route; the optimal nutrient levels in DCAP and HP promote growth, whereby increasing the sink strength which induces positive feedback on photosynthesis, thus ultimately enhancing growth (Ruiz-Vera et al., 2017). Interestingly, the recorded reduction in CAP biomass accumulation was more dramatic than the outcome of cumulative yield. This indicates that fruit production is a multifaceted process influenced by more factors than just biomass. More importantly, it reflects the trade-off between vegetative growth and fruiting in terms of photosynthate allocation and nutrients partitioning (Kim et al., 2019). On the other hand, the similarity or occasionally better performance of DCAP over HP may be associated with the possible presence of growth-promoting microbes and/or of specific dissolved organic molecules in the RAS solution (Rurangwa and Verdegem, 2015; Schmautz et al., 2017; Grunert et al., 2020). It remains uncertain whether microorganisms present in the fish water or within the rhizosphere of DCAP plants, played a role in enhancing plant growth or nutrient uptake (Monsees et al., 2019; Delaide et al., 2019). Notably, in this context, one might hypothesize that the external fertilizers added to DCAP amplified these effects, given that CAP plants, despite receiving the same fish water, did not exhibit a similar response. With the addition of insect meal to the tilapia feed we may hypothesize that the microbial load is large; to unravel such interactions, our group is in the process of estimating and identifying the microbial presence in the system components, which will provide conclusive answers.

The inferiority of coupled aquaponics in terms of growth and productivity has been extensively attributed to the poor leaf nutritional state, which reflects the deficiencies of important nutrients in fish water (Lobanov et al., 2021; Rodgers et al., 2022; Tellbüscher et al., 2025). This was explicitly illustrated in a series of laboratory-scale experiments following the “minimal inputs approach”, where the sole addition of K in the irrigation water resulted in manifold increases in growth performance in lettuce, spinach and spiny chicory compared to non-amended fish water (Tsoumalakou et al., 2022, 2023b, 2023a). In the present study, CAP plants suffered from extremely low K levels and secondly low [P], whereas the leaf N content was decreased only towards the end of the experiment (**Figure 7**). The source of N, P and K in the CAP irrigation solution is the fish excretions, which consist of 30-65% nitrogen, 40% phosphorus and nearly zero K, because fish feed is seriously depleted in K and fish consume and use most of it (Schneider et al., 2005). This explains the suboptimal levels of K typically found in aquaponics systems, resulting in leaf deficiencies (Roosta, 2014a; Harika et al., 2024). Both K and P play crucial roles in many biological processes, affecting plant metabolism, the performance of the photosynthetic apparatus, protein synthesis and osmotic potential (Roosta, 2014b; Patel et al., 2022). Therefore, their sufficient levels in the leaf tissue are important for the optimization of crop growth, yield and quality of the products (John et al., 2022). Contrarily to CAP, the crop growth in DCAP was similar to HP. Corroborating this result, a previous experiment in the pilot-scale experimental setup like the one presented here reported that DCAP-treated basil showed a trend toward enhanced biomass production compared to HP, though the differences were not statistically significant (Mourantian et al., 2023). Analogous results were obtained for lettuce growth under the DCAP treatment, in which all the measured growth parameters were similar to HP (Chandrou et al., 2024). Additionally, Roosta (2014b) working with K foliar application found that levels similar to HP and DCAP of the present study considerably increased leaf K content and subsequently improved biomass gain in all tested aquaponics species. In terms of productivity output, Aslanidou et al. (2024) reported a 40% yield reduction in CAP cucumber, which is close to our findings. However, in their results a superiority of DCAP compared with HP was evident for basil and parsley yield, while in cucumber the levels were similar.

Nutrient status is a key factor that shapes both the growth response and the functional performance of plants, especially in high-nutrient demanding crops such as cucumber. The chlorophyll *a* fluorescence determined *in vivo* is a valuable tool of non-destructive assessment of the functioning and efficiency of the photosynthetic apparatus. It can give detailed information on energy fluxes and the performance of partial processes along the electron transport chain of PSII, together with indications of PSI function (Strasser et al., 2004; Chondrogiannis and Grammatikopoulos, 2016). The fluorescence profile of DCAP plants corresponded to increased energy fluxes per reaction center accompanied by enhanced PSII performance indices, indicating a well performing photosynthetic apparatus, which also was obvious in HP plants. On the contrary, CAP leaves experienced a substantial increase in thermally dissipated energy per active RC (DIo/RC), which along with the increasing limitations of PSI-related yields, collectively resulted in decreased photochemical efficiency compared to DCAP and HP. Nutrient deficiencies, such as those observed in the CAP plants, are well-documented to induce alterations in PSII photochemistry, analogous to those of cucumber in the present study (Larbi et al., 2006; Tsoumalakou et al., 2022). Using the same experimental setup as the present, Chandrou et al. (2024) examined the combined effects of aquaponics and biostimulants on lettuce and reported analogous declines in PI_TOTAL_ and upward trends in DIo/RC in the CAP plants, corroborating our results. Roosta (2014a) observed similar results in aquaponics basil ascribing them to K, Fe and Mn deficiencies, while Jardim et al. (2021) attributed lower PSII quantum yield and enhanced non-photochemical quenching to K deficiency in opuntia leaves.

The above-mentioned superiority of DCAP in terms of photochemical efficiency was also depicted in the gas exchange parameters. The net photosynthetic rate of DCAP leaves was consistently higher than CAP and HP, yet statistically significant differences were recorded only with CAP. The latter presented a reduction of A_N_, though it never exceeded 20% compared to HP. Analogous profile of variations during the experiment was evident in Tr, while the iWUE did not show any consistent trend during the measurement period. The stomatal conductance of CAP plants decreased significantly (except for D20) compared to the other two treatments. Several works on various crops correlate this decline, among others, to K deficiency (Singh and Reddy, 2018; Javornik et al., 2023) which is also confirmed by the foliar K concentration in CAP. K homeostasis is a crucial parameter for stomatal regulation, therefore the pattern of g_s_ fluctuations in CAP may indicate stomatal limitations to photosynthesis (Hasanuzzaman et al., 2018).

The overall photosynthetic performance, as inferred from gas exchange and chlorophyll fluorescence assessments, is more indicative of down-regulation of relevant processes than damage to the photosynthetic apparatus. The indications supporting this hypothesis include the moderate reductions in A_N_ (maximum 20%) and the similarly modest decline of certain efficiency indices in the fluorescence profile. Also corroborating this hypothesis are the results of our previous aquaponics research, in which the focus shifted from yield results to underlying physiological mechanisms, especially the detailed analysis of photosynthetic attributes (Tsoumalakou et al., 2022; Mourantian et al., 2023; Chandrou et al., 2024). It seems that CAP plants, through this down-regulation, modify their metabolism levels to acclimate to the low nutrient availability in their irrigation solution and consequently their sub-optimal foliar nutrient content. This acclimation does not negatively impact the structure and function of the photosynthetic apparatus, whereas it does severely affect growth and yield of CAP plants. The mechanisms underlying the growth effects are complex and multifaceted (Tang et al., 2015; Hasanuzzaman et al., 2018). Indicatively for K, sub-optimal levels in leaves may be involved in the perturbation of photosynthate partitioning and consumption (Patel et al., 2022), as well as the maintenance of optimal turgor pressure, which is crucial for cell elongation and leaf area expansion in young leaves (Pettigrew, 2008).

DCAP, on the other hand, showed comparable or even higher levels of all the measured parameters than HP, indicating its increased potential for sustaining high productivity while using significantly less fertilizer. Collectively, these results point to a more sustainable cropping systems than the conventional hydroponics. The 74% increase in FUE of DCAP cucumber compared to HP suggests that DCAP cucumber cultivation optimizes both the environmental footprint and the economic aspect of fertilizer consumption. Towards this direction are also the findings of Pinho et al. (2024) who worked with BSF meal substitution in tilapia feed in co-cultivation with lettuce. They did not estimate FUE, however, they reported a 32% reduction in fertilizer consumption compared to HP. Similarly, they do not determine WUE but their estimations of the volume of water used per plant per day corroborate our results of similar WUE, reporting same levels between DCAP and HP.

An evaluation of the commercial feasibility and scalability of the tri-trophic system, particularly in economic and technical contexts, is beyond the scope of this study. However, some important and relevant aspects of the current approach can be identified. The insect-based feed, found to effectively support fish growth, could easily be implemented in the numerous industrial facilities for insect farming and production of insect-based feed that have emerged worldwide over the past decade. This, alongside the increasing acceptance of insects as feed by the European Union, as reflected in the approved use for fish, husbandry animals and pets feed, ensures the commercial feasibility of their production and facilitates their integration into a large-scale tri-trophic system such as the one proposed here. Another potential advantage that could encourage commercial adoption is the substantial economic savings from reduced fertilizer use, as demonstrated in this study. Finally, with regard to the scalability of this system is ensured by the occurrence of commercial-scale aquaponics, taking advantage of the versatile character of system, the good performance of which was proven in the pilot-scale aquaponics greenhouse used in the present study.

Designing sustainable food production systems is part of Sustainable Development Goal 2 (SDG 2), which aims to achieve “zero hunger” and was established by the United Nations in 2015. More specifically, it is part of Target 2.4, which concerns the implementation of sustainable food production and resilient agricultural practices. The circular tri-trophic system presented in this study can contribute to SDG 2 because it produces nutritious and healthy food (crops and fish), encourages local production, and promotes the recycling of organic waste and by-products and more sustainable use of land and water. Given the above, and its additional important trait of high self-sufficiency, the system can be considered a model of sustainable agriculture in practice. The latter, in a broader perspective, is not only an environmental and economic issue, but also a subject of international policy regarding its potential to tackle environmental risks.

## 5 Conclusions

The circular tri-trophic production system presented in this study incorporates plants, fish and insects in a single loop, where the waste or by-product of one species is turned into a resource for feeding the other. The comprehensive evaluation of physiological, growth and yield performance of crops subjected to the three treatments indicated that DCAP was the best performing one. The high efficiency of the photochemical and photosynthetic processes, along with increased biomass accumulation and yield similar to the control HP point to a productive system that overcomes the limitations that impeded growth of CAP. The latter resulted in lower growth and yield; however, the physiological data indicate a down-regulation of processes to acclimate to the sub-optimal nutritional state of leaves, rather than a damage to the photosynthetic apparatus. Finally, the increased FUE of DCAP and the similar WUE compared to HP, highlight that DCAP is an efficient production system with increased sustainability compared to the conventional hydroponics. The overall performance of the tri-trophic system demonstrates its feasibility and applicability on the large, commercial scale. With the incorporation of decoupled aquaponics, this circular system enables the simultaneous production of high-demand crops, such as cucumbers and fish fed with sustainably produced insect protein. At the system-level, it has the potential to reduce the environmental impact of food production systems and could therefore be regarded as a paradigm of sustainable agriculture.

## Data Availability

The original contributions presented in the study are included in the article/**Supplementary Material**. Further inquiries can be directed to the corresponding author.
