# Supplementary material for: A circular tri-trophic system incorporating plants, fish, and insects turns waste into a resource: case study with the cultivation of cucumber
Source: Front Plant Sci. 2025 Oct 14;16:1638443. doi: 10.3389/fpls.2025.1638443 (PMC12560244; doi:10.3389/fpls.2025.1638443)
Supplement: Supplementary file 1 [file DataSheet1.docx]

**Supplementary Material**


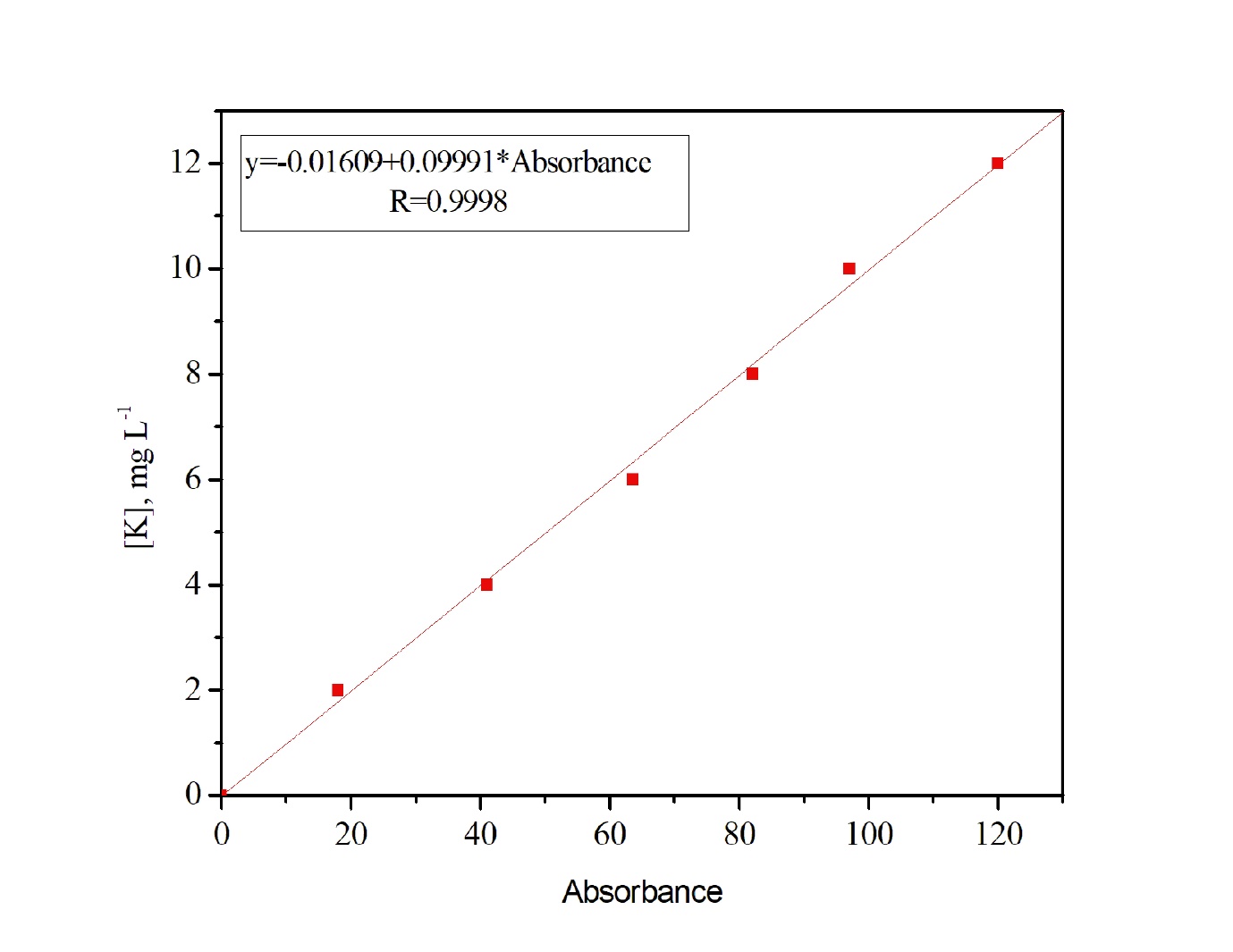


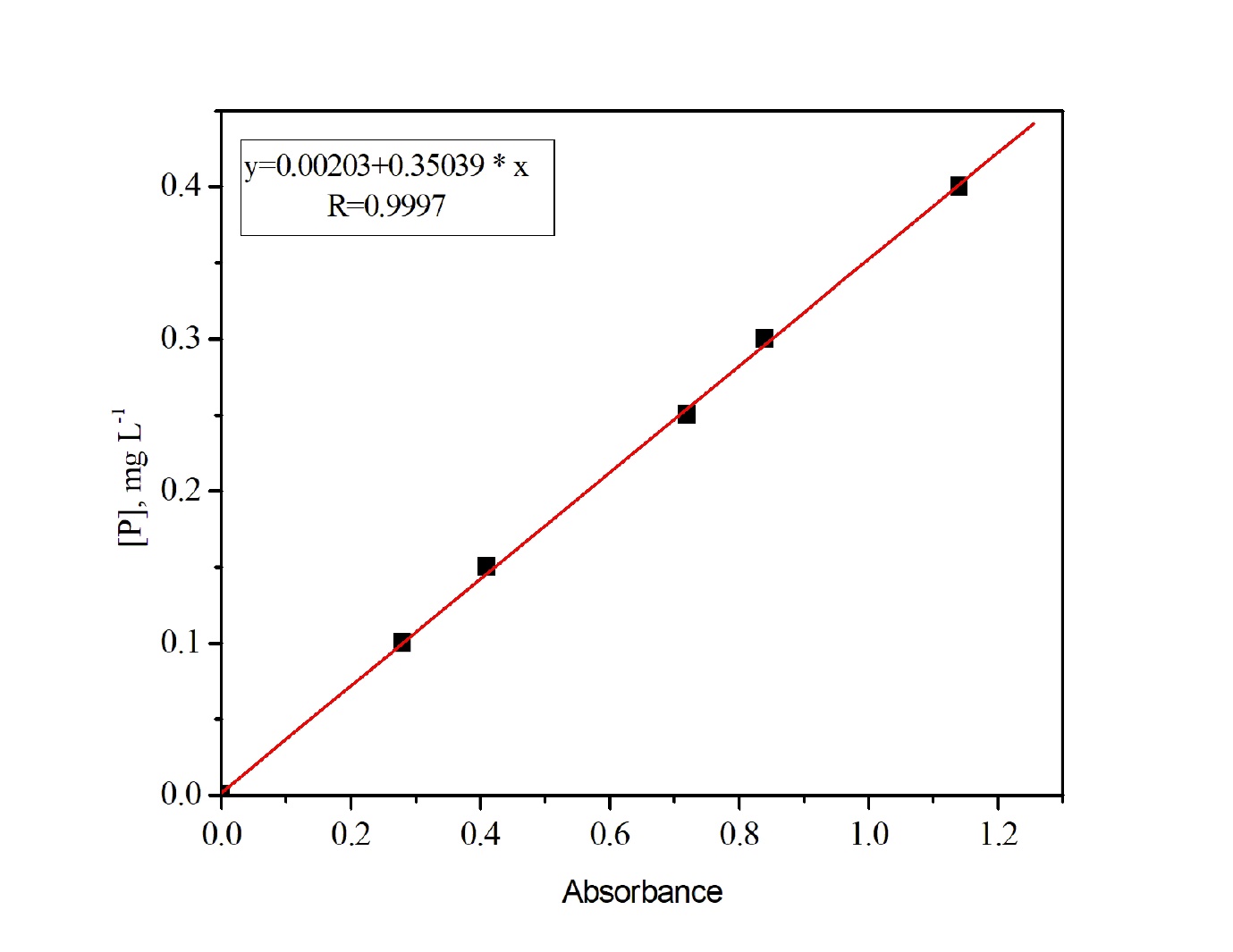


Figure S1. Calibration curves for the determination of [K] (upper graph) and [P] (lower graph) in leaves of cucumber plants.
